# Simultaneous measurement of multiple variant-specific SARS-CoV-2 neutralizing antibodies with a multiplexed flow cytometric assay

**DOI:** 10.3389/fimmu.2022.1039163

**Published:** 2022-11-25

**Authors:** Hong Liu, Stephen Varvel, Ge Chen, Joseph McConnell, Rebecca Caffrey, Marzena Galdzicka, Shahrokh Shabahang

**Affiliations:** Aditxt, Inc., Richmond, VA, United States

**Keywords:** COVID-19, SARS-CoV-2, neutralizing antibodies, omicron subvariants, flow cytometry, clinical

## Abstract

**Introduction:**

Neutralizing antibodies (NAbs) have been recognized as surrogates of protection against SARS-CoV-2; however, the emergence of variants/subvariants escaping neutralization suggests that laboratory assessments of NAbs against the ancestral/wild type (WT) antigens likely overestimate the degree of protection.

**Methods:**

A novel flow cytometry-based multiplex test system was developed for the simultaneous detection of NAbs of multiple SARS-CoV-2 variants. SARS-CoV-2 antibodies (Abs) including IgG, IgM, IgA isotypes were measured in the same system. Samples from negative, convalesced, vaccinated, boosted, and breakthrough infection (BTI) populations were tested for both NAbs and Abs.

**Results:**

NAbs induced by WT showed neutralization activity that correlated strongly to all variants (R^2^ > 0.85) except omicron BA.1/BA.2 (R^2^ <0.50). Two doses of vaccine elicited very little protective immunity against BA.1/BA.2, though a booster dose significantly improved NAbs for all variants. NAbs/Abs increased more following BTI than after a booster, suggesting that hybrid immunity (vaccination + natural immunity) was more robust to all variants including BA.1/BA.2. BTIs occurring in the omicron era led to stronger NAb responses against BA.1/BA.2 than did older BTIs. In all comparisons, the RBD antigens demonstrated greater differences between WT and BA.1/BA.2 than the spike antigens.

**Discussion:**

Taken together, we demonstrated that both Ab and NAb against multiple SARS-CoV-2 variants/subvariants can be reliably detected on the same multiplex platform. Distinguishing NAbs to the appropriate antigenic target of prevalent variants offers the best correlate of protection and aids individual decisions about the appropriateness and cadence of vaccine boosters and other exposure mitigation strategies.

## Introduction

Efforts to assess individuals’ protection against SARS-CoV-2 infection have been challenged by the emergence of new variants of the SARS-CoV-2 virus which display different degrees of infectivity, pathogenicity, and escape from immune defenses established by vaccination or previous exposure. The ancestral/wildtype (WT) form of the virus has been followed by a series of “variants of concern”, each displaying slightly different clinical characteristics (1).

Pre-omicron, all variants retained enough similarity to the SARS-CoV-2 WT virus to be recognized and neutralized by the immune system. Specifically, antibodies developed after vaccination or previous exposure to an earlier form of the virus largely retained the ability to neutralize most variants (i.e., block the primary mechanism the virus uses to infect human cells by interfering with binding of the spike protein to human ACE-2 receptor), though small differences in effectiveness were noted. This antibody-mediated neutralization has been regarded as a “correlate of protection” (2–4), allowing for tests of neutralizing antibody activity (NAb) to be used as an estimate of an individual’s current level of protection against SARS-CoV-2 infection.

The omicron BA.1 (B.1.1.529.1) variant, which was first identified in November 2021 and rapidly spread around the world to become the dominant form of the virus (5), was the first variant shown to substantially escape existing antibody defenses. In both live virus plaque reduction and ACE-2 binding inhibition assays, several groups demonstrated that antibodies induced by vaccines or by prior SARS-CoV-2 infection had a significantly reduced ability to neutralize omicron (6–12).

Subsequently, a second omicron variant BA.2 (B.1.1.529.2) was identified that appears to be as effective as BA.1 in escaping vaccine-induced NAbs (13, 14). By late March 2022, omicron BA.2 had already overtaken BA.1 as the dominant strain in the U.S. (5). Early indications are that omicron infection elicits a NAb response that is effective at neutralizing both BA.1 and BA.2 (14, 15). Subsequent omicron subvariants (e.g., BA.4/5) are now spreading rapidly, though the degree to which they are neutralized by antibodies elicited by earlier omicron infection is not yet clear.

Methods to rapidly identify variants displaying substantial NAb evasion and to incorporate relevant target antigens into clinical tests are needed to provide accurate and timely assessment of individual immune status against variants likely to be encountered. We have established a novel multiplex high-throughput flow cytometry-based assay that estimates neutralizing activity against antigen from multiple variants simultaneously. Here we assess the neutralizing efficacy of plasma from 6 cohorts of naïve, COVID-19 convalesced (non-vaccinated), twice vaccinated, and boosted individuals against 8 different SARS-CoV-2 variants/subvariants. We further compared neutralizing capacity to WT, BA.1, and BA.2 RBD and spike antigens in subjects who experienced breakthrough infections in the pre- and post-omicron eras (5).

## Methods

### Study design

In order to determine the degree to which antibodies elicited by vaccination or prior SARS-CoV-2 infection neutralize different variants, we generated a bead set that contained the receptor binding domain (RBD) antigen from SARS-CoV-2 WT, alpha, beta, delta, epsilon, gamma, and omicron BA.1 and BA.2 variants. Neutralizing activity (% inhibition of ACE-2 binding) and RBD IgG, IgM, and IgA antibody levels were measured for each clinical cohort. Based on these results we prepared two new sets of beads to compare NAbs and Ab profiles for either RBD or spike protein of WT, BA.1, and BA.2 and compared NAb and Ab levels among cohorts. Finally, NAb and Ab of a subset of the vaccinated group who experienced a breakthrough infection (BTI) either before (occurring before Dec 5, 2021) or during (occurring after Dec 30, 2021) the omicron era were compared.

### SARS-CoV-2 neutralizing antibody testing procedure

In brief, neutralizing antibody strength was determined with a novel multiplex flow cytometry based competitive inhibition assay for the measurement of neutralizing antibodies to SARS-CoV-2 in human plasma samples. In a 96 well round bottom plate, 20 µL plasma or serum was incubated with 5 µL RBD-Microparticle mix, 20 µL Biotinylated ACE-2 (made in-house) and 5 µL SA-PE (Cat No. 016-110-084, Jackson Research, West Grove, PA 19390) for 60 minutes. After 2 washes with 150 µL of 1% BSA/PBS, the microparticles were acquired on a BD FACSLyric™ Flow Cytometer. The NAb % was calculated by the following formula: NAb (%) = [1% BSA/PBS (MFI) – Sample (MFI)]/1% BSA/PBS (MFI) X %. Percent ACE-2 binding inhibition values were converted to International Units per milliliter (IU/mL) based on a polynomial function determined by comparison to a standard curve with known neutralizing antibody concentrations (human NIH SARS-CoV-2 serology standard, Lot # COVID-NS01097, characterized and made available by Frederick National Laboratory for Cancer Research [FNLCR], Frederick, Maryland, USA).

### SARS-CoV-2 antibody testing procedure

SARS-CoV-2 antibodies of 3 isotypes (IgG, IgM, and IgA) to either 8 RBD (WT, alpha, beta, delta, epsilon, gamma, and omicron BA.1 and BA.2) or 3 RBD or Spike (WT, omicron BA.1 and BA.2) SARS-CoV-2 antigens were assessed with violet- fluorescent polystyrene beads of different intensities, each conjugated to a separate SARS-CoV-2 viral antigen. The 5 µL bead mixture was incubated with 50µL human blood plasma (1:100 dilution) in a 96-well plate, in which SARS-CoV-2 antibodies present in the sample bind to the antigen-conjugated beads. Unbound antibodies were removed by wash buffer, and a secondary detection antibody mixture was added containing fluorochrome-conjugated antibodies specific for human IgG, IgM, and IgA. The median fluorescence intensity (MFI) for each species was measured on a BD FACSLyric flow cytometer and relative fluorescent intensities were compared to a standard curve to quantify the amount of anti-SARS-CoV-2 antibody present in the sample.

### Preparation of RBD and spike protein microparticles

SARS-CoV-2 RBD wild-type protein (WT) was purchased from ExonBio (Cat No.19CoV-S120, San Diego, CA 92121). All SARS-CoV-2 RBD variant proteins were purchased from ACROBiosystems (RBD-α, Cat No. SPD-C52Hn; RBD-b, Cat No. SPD-C52Hp; RBD-ɗ, Cat No. SPD-C52Hh; RBD-ε, Cat No. SPD-C52He; RBD-ɣ, Cat No. SPD-C52Hr; RBD-o-BA1, Cat No. SPD-C522e; RBD-o-BA2, Cat No. SPD-C522g; Newark, DE 19711. SARS-CoV-2 whole spike wild-type protein was made in-house (Lot No. 051421) and spike omicron proteins were purchased from ACROBiosystems (Spike-o-BA1, Cat No. SPN-C52Hz; Spike-o-BA2, Cat No. SPN-C5223; Newark, DE 19711). The RBD and Spike proteins were conjugated onto polystyrene microparticles with different fluorescence IDs (Spherotech, Lake Forest, IL 60045) by the Two-Step EDC (Pierce biotechnology, Rockford, IL 61105) conjugation protocol. The same sets of coated beads were used for the NAb and Ab assays.

### Study population

Subjects included in this analysis were primarily a subset of study participants enrolled in a prospective cohort study evaluating SARS-CoV-2 immune responses (NCT05379478), approved by WCG IRB (IRB #20202768). At successive study visits at an Aditxt immune monitoring center in Richmond, VA (RVA) or Mountain View, CA (MV), subjects with a history of SARS-CoV-2 infection or vaccination provided blood (EDTA plasma) and saliva samples for determination of antibody profiles and current COVID-19 status, as well as a limited set of clinical and demographic information. Samples were stored at -80^°^ until testing.

A second source of samples was the Stanford Blood Center (SBC, Palo Alto, CA), which provided a set of COVID-19 negative samples (Negative, group 1) collected prior to 2017, as well as a set of samples from convalesced (non-vaccinated) patients (Convalesced, group 2) collected between Jan – May 2020. Although demographic information (i.e., sex and age) was not available for these two SBC cohorts, the cohorts were included in this analysis as they provide information complementary to the samples obtained through the primary study. Specifically, the negatives can be considered “true negatives” as they were collected before the pandemic while the RVA negatives were collected during the pandemic (defined as no known SARS-CoV-2 exposure or vaccination and no detectable SARS-CoV-2 antibodies). Differences were noted between the convalesced SBC and RVA cohorts that warranted including both (as described below). The RVA convalesced subjects (Convalesced, group 1) were non-hospitalized volunteers with very mild to moderate symptoms occurring between November 2020 **-** September 2021, whereas the SBC convalesced were collected while the WT form of the virus was prevalent and were presumably seeking treatment (i.e., more severe symptoms). No information on sex or age was available for samples obtained from SBC, and age data were missing from 3 subjects in the vaccinated and boosted groups. Characteristics of participating subjects are shown in **Supplemental Table 1**, and a brief description of each cohort is provided here.

Negative Group 1 (SBC):

Samples (N=15) were collected before the outbreak of the pandemic (before 2017) and tested negative for SARS-CoV-2 antibodies and neutralizing antibodies (defined as no known SARS-CoV-2 exposure or vaccination and no detectable SARS-CoV-2 antibodies).

Negative Group 2 (RVA):

Samples (N = 22) were collected after the outbreak of the pandemic (2021-2022) and were negative for SARS-CoV-2 antibodies and neutralizing antibodies.

Convalescent Group 1 (RVA):

Samples (N=18) were collected from non-hospitalized volunteers with very mild to moderate symptoms with PCR-confirmed positive infections between November 2020 and September 2021.

Convalescent Group 2 (SBC):

Samples (N=30) were collected from patients who recovered from COVID-19 between January 2020 and May 2020; All patients developed severe COVID-19 symptoms and were confirmed positive by FDA EUA-approved RT-PCR for SARS-CoV-2.

Vaccinated Group (RVA + MV):

Samples (N=46) were collected from patients with no COVID history or positive NP antibodies 1 week – 4 months after their second dose of the Pfizer or Moderna vaccine.

Booster Group (RVA + MV):

Samples (N=29) were collected from patients with no COVID history or positive NP antibodies 1 week – 4 months after their third (booster) dose of either Pfizer or Moderna vaccine.

### Statistical analysis

All analyses were performed in Prism (v9; GraphPad Software, San Diego, CA, USA). Two-way ANOVAs were used to assess the effects of Cohort (between-subject) and Variant (within-subject) on laboratory measures. F statistics for significant Cohort x Variant interactions are presented in **Supplemental Table 2**. Dunnett’s multiple comparisons were used to determine individual differences, with a family-wise alpha threshold of 0.01. Reported p values for each comparison are multiplicity adjusted.

## Results

**Figure 1:**
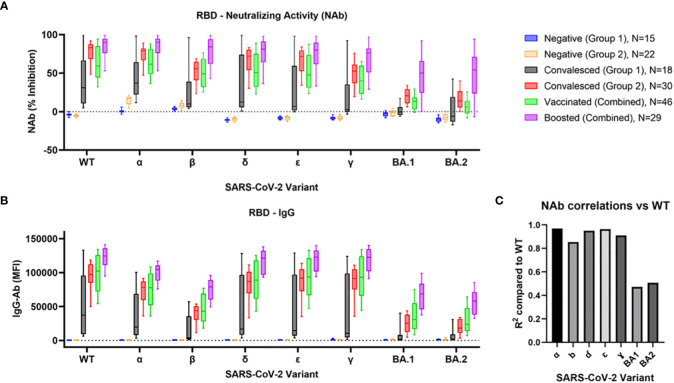
Comparison of RBD Nab and Ab to BA.1, BA.2, and prior SARS-CoV-2 variants. Variant-specific neutralizing antibody activity **(A)** and IgG antibodies **(B)** were determined in 6 cohorts: 1) COVID-naïve non-vaccinated negative controls collected before the beginning of the pandemic [Negative (Group 1)], 2) negative controls collected during 2021 [Negative (Group 2)], 3) a group of convalesced subjects with predominantly mild symptoms [Convalesced (Group 1)], 4) a second group of convalesced subjects from Stanford Blood Center [Convalesced (Group 2)], as well as groups of vaccinated (Vaccinated), and vaccine boosted (Boosted) individuals. Correlations between NAb activity at each variant compared to wildtype are shown **(C)**.

NAb activity of each cohort against the RBD of eight different SARS-CoV-2 variants is shown in **Figure 1A**. Within each variant, a similar pattern of NAb activity was observed across all cohorts. The Convalesced Group 1 (with milder symptoms) showed only modest activity which was significantly elevated against only the WT (p<0.01), alpha (p<0.001), delta (p<0.01), and epsilon (p<0.05) variants. The Convalesced Group 2 (with more severe disease) and the vaccinated group showed more robust NAb activity to all variants, though activity to BA.1 and BA.2 was lower than all pre-omicron variants (p<0.0001). The highest levels of NAbs were seen in the boosted cohort, which were significantly higher than vaccinated subjects for all variants (p<0.0001). When all the cohorts were combined (N=167) correlations comparing NAbs for each variant to WT resulted in R^2^ values > 0.90 for each variant except for beta (R^2^ = 0.85), BA1 (R^2^ = 0.47), and BA.2 (R^2^ = 0.51; **Figure 1C**).

IgG antibody levels (MFI) for each cohort and variant are shown in **Figure 1B**. The pattern of individual multiple comparisons closely mimicked that of the NAbs. Across all variants and cohorts (N = 1272), IgG levels were strongly correlated with NAb activity (R^2^ = 0.79, p<0.0001). Results for corresponding IgM and IgA levels are shown in **Supplemental Figure 1A**. Of note, only the Convalesced Group 2 showed robust increases in IgM and IgA to all variants except beta, BA.1, and BA.2 (p<0.0001).

**Figure 2:**
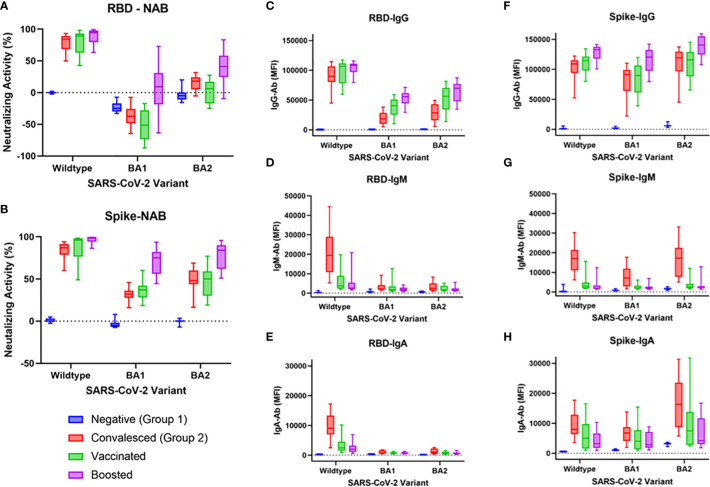
NAb activity and IgG, IgM, and IgA antibodies to WT, BA1, or BA2 spike or RBD antigen in negative, convalesced, vaccinated, and boosted subjects. NAbs were significantly lower for BA1 and BA2 compared to wildtype in convalesced, vaccinated, and boosted groups when either RBD antigen **(A)** or spike antigen **(B)** were used. Boosted groups displayed higher cross-neutralization to BA.1 and BA.2 than both convalesced and vaccinated groups **(A, B)**, though less than to WT. These differences were more pronounced when RBD antigen was used compared to spike, demonstrating greater variant-specificity with the RBD beads. IgG levels to spike antigen **(F)** were higher than those to RBD **(C)**. IgM **(D, G)** and IgA **(E, H)** were highest in the convalesced group (compared to vaccinated or boosted) and were higher to spike BA.1/BA.2 than RBD BA.1/BA.2.

Results of assessment of NAbs, IgG, IgM, and IgA Ab levels against either RBD or Spike WT, BA.1, and BA.2 are shown in **Figure 2**. As expected, the convalesced, vaccinated, and boosted cohorts all demonstrated robust NAb levels compared to negative controls for both WT RBD and WT Spike Protein (all comparisons p<0.0001, **Figure 2A**). Surprisingly, the BA.1 RBD (but not BA.1 spike) showed significant “negative inhibition” (i.e., ACE-2 binding appeared to be enhanced compared to controls) for the negative, convalesced, and vaccinated (all p <0.0001), but not the boosted cohorts. A small but significant increase in NAbs compared to negative was seen for the BA.2 RBD in the convalesced (p<0.01) and boosted (p<0.0001) but not vaccinated groups (p=0.63), while the boosted group was elevated compared to vaccinated (p<0.001). With the Spike antigen (**Figure 2B**), the convalesced and vaccinated groups showed modest increases in NAbs compared to negatives for both BA.1 and BA.2 (p<0.0001), and the levels in the boosted cohort was significantly higher than those in the vaccinated group (p<0.001). Convalesced, vaccinated, and boosted cohorts all showed significantly lower NAb levels against BA.1 and BA.2 compared to WT (p<0.0001 for all comparisons), and in all cohorts NAbs were higher for Spike BA.1 and BA.2 compared to RBD (p<0.0001).

RBD IgG and Spike IgG levels are shown in **Figures 2C, F**, respectively. As shown in **Figure 2C**, WT RBD IgG levels did not differ between convalesced, vaccinated, and boosted cohorts. BA1 and BA2 RBD IgG levels were lower than WT for each cohort other than negatives (p<0.0001). For BA1, IgG levels in the vaccinated group were slightly higher than those in the convalesced (p<0.05), while boosted levels were higher than convalesced (p<0.0001) and vaccinated (p<0.01). For BA2, boosted levels were also higher than both convalesced (p<0.0001) and vaccinated (p<0.05). For all combinations, IgG was higher for Spike than for RBD (p<0.0001).

As shown in **Figures 2D, G**, only the convalesced cohort had elevated IgM against WT (p<0.0001), while IgM against Spike (but not RBD) was also elevated to BA.1 and BA.2 (p<0.0001). A similar pattern was observed for RBD IgA and Spike IgA. As shown in **Figures 2E, H**, all three cohorts (convalesced, vaccinated, and boosted) had higher IgA than negatives to both RBD and Spike (at least p<0.05). Convalesced IgA levels were also higher than vaccinated or boosted for RBD WT (p<0.001) and for Spike WT, Spike BA.1, and Spike BA.2 (at least p<0.05).

**Figure 3:**
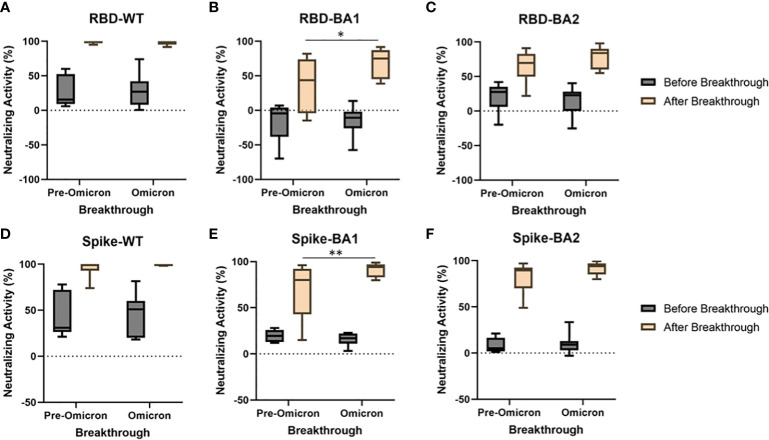
A subset of vaccinated individuals experienced breakthrough infections during the study. Breakthrough infections were separated into those likely to be pre-omicron (symptom onset before Dec 5, 2021, N=6) and those likely to be omicron (symptom onset after Dec 30, 2021, N=11). Breakthroughs occurring during both eras produced near maximal increases in WT Nabs using RBD **(A)** and spike **(D)**. Increases in Nabs to BA1 **(B, E)**, but not BA2 **(C, F)** were significantly greater when the breakthrough occurred in the omicron era compared to when the breakthrough occurred pre-omicron for both RBD (p<0.05) and spike (p<0.01). *p<0.05, **p<0.01.

As shown in **Figure 3**, breakthrough infections led to robust increases in NAbs to WT, and smaller, though substantial, increases to BA.1 and BA.2 (p<0.0001 for all before vs. after breakthrough comparisons). The variant involved with these infections was not identified empirically, but the marked spread of omicron during the month of December 2021 allows a reasonable presumption to be made regarding pre-omicron and omicron infections based on their prevalence reported through the CDC’s surveillance program (e.g., omicron infections were estimated to have increased from <1% to >95% of cases during this period, 5). For both WT RBD (**Figure 3A**) and WT Spike (**Figure 3D**), both eras of breakthrough produced near maximal NAb increases, even higher than those seen in the boosted groups shown in **Figure 2** (p<0.0001). Significantly higher BA.1 NAbs were seen following an omicron-era breakthrough compared to pre-omicron breakthroughs (p<0.05 and p<0.01, respectively). Increases in BA.2 NAbs following breakthrough were not different between pre-omicron and omicron era breakthroughs. RBD IgG, IgM, and IgA antibodies for WT, BA.1, and BA.2 are shown in **Supplemental Figure 2**. Breakthrough infections led to significant increases in IgG and IgA, but not IgM, for all three variants. There were no differences detected based on whether the breakthroughs were pre-omicron or likely omicron.

**Figure 4:**
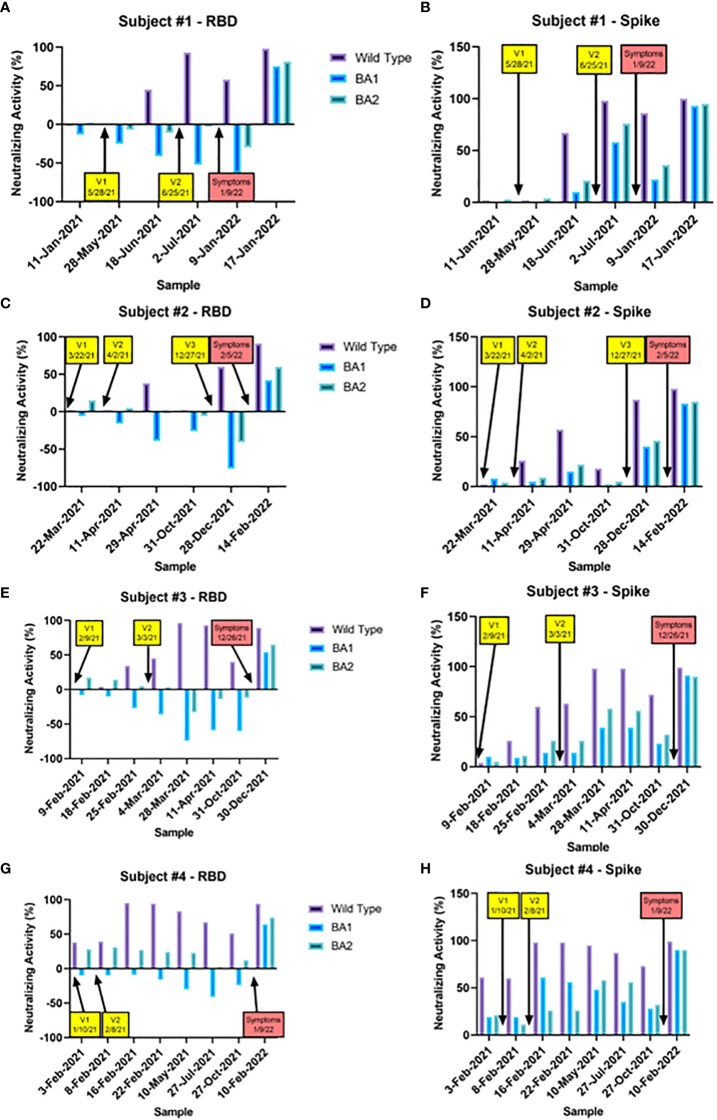
Nabs for RBD and Spike WT, BA.1, and BA.2 are shown for a set of samples taken from four representative vaccinated subjects who experienced a breakthrough infection during the omicron era. The timing of each vaccination (yellow boxes) and the onset of symptoms (red boxes) are indicated with arrows. In all four cases, vaccination robustly increased WT Nabs to RBD and Spike to a similar degree. BA.1 and BA.2 Nabs were not increased at all by vaccination (in fact were negative) when the RBD antigen was used **(A, C, E, G)**, and increased only slightly with the spike antigen **(B, D, F, H)**. In contrast, omicron breakthroughs led to increases in Nabs for WT, BA.1, and BA.2 with both RBD and Spike. In all four cases, RBD BA.1 and BA.2 Nabs were the lowest at the time of the breakthrough, suggesting they were they best indicator of infection risk.

Variant-specific NAbs from a representative set of four vaccinated subjects who experienced an omicron breakthrough infection (likely BA.1/BA.2 based on the date of infection) are shown in **Figure 4**. In all four cases, vaccination produced moderate to strong NAb responses to both WT RBD and Spike. In contrast, vaccines produced virtually no BA.1 or BA.2 RBD NAbs, and a low to moderate level of BA.1 and BA.2 Spike NAbs. As seen in the breakthrough experiments shown in **Figure 3**, greater NAbs to BA.1 and BA.2 were seen when the Spike antigen was used compared to RBD. The fact that BA.1/BA.2 RBD NAb levels were the lowest at the time of the breakthrough suggests they were a better indicator of the risk of infection than the BA.1/BA.2 Spike NAbs, which were still suggestive of some protection.

## Discussion

One consequence of the COVID-19 pandemic has been an increase in interest in assessment of an individual’s level of protection against the SARS-CoV-2 virus. Our results demonstrate that SARS-CoV-2 NAb and Ab against WT and other variants can be reliably detected on the same multiplex platform, supporting its clinical utility. Importantly, NAbs induced by vaccination or prior infection with a pre-omicron variant showed WT neutralization activity that correlated strongly with all variants except BA.1/BA.2. This observation is consistent with the increased rate of breakthrough infections that has been reported since the emergence of the omicron variants. In contrast, breakthrough infections in the omicron-era led to stronger increases in omicron neutralization than did pre-omicron infections. Furthermore, we found that this multiplex platform is more variant specific and clinically relevant when the RBD antigen is used compared to the whole Spike protein.

Neutralizing antibodies, which block the interaction between the SARS-CoV-2 Spike protein and human ACE-2 receptor and thus prevent infection, have been generally acknowledged as a useful correlate of protection. For example, NAbs have been shown to be mechanistically protective in animal models (16) and neutralizing monoclonal antibody therapies have been successful at improving clinical outcomes (17). Furthermore, in pivotal COVID-19 vaccine trials, the degree of elicited NAbs was correlated with efficacy in reducing subsequent infections (3, 18, 19). Since then, NAbs have been used to predict vaccine efficacy and have informed strategies of appropriateness and cadence of vaccine boosters. However, the gold standard tests of neutralizing activity (virus neutralization bioassays) require live SARS-CoV-2 virus in a biosafety level 3 facility, limiting their use to research purposes. Even pseudovirus-based assays, which use spike protein from SARS-CoV-2 that has been incorporated into less pathogenic viruses, require a biosafety level 2 laboratory and are not practical for high-scale clinical application. Surrogate virus neutralization tests that assess the ability of patient serum to inhibit RBD – ACE-2 binding, such as the assay presented here, correlate well with pseudo-virus assays, are much simpler to run, and are scalable for wide availability.

Tests for neutralizing activity are naturally focused on the spike protein (and particularly the RBD) and are thus susceptible to differences among the emergent variants, as many of the defining mutations cluster around the Spike/RBD region of the virus. Assessing neutralizing activity with tests designed for the WT form of SARS-CoV-2 will likely not be appropriate for variants that largely escape those antibodies. The multiplexing capabilities of our assay which enables the simultaneous assessment of multiple targets (e.g., variants or other viruses) was a deliberate design to allow surveillance of new variants of interest and rapid deployment of new clinical tests that target and incorporate variants of greatest interest. In our initial experiments, only the BA.1 and BA.2 subvariants showed significant antibody escape (i.e., <50% correlation with WT NAbs) resulting in our next assay to include 3 beads targeting WT, BA.1, and BA.2. If future variants demonstrate similar degrees of escape from both WT and BA.1/BA.2, modifications will be made to include those antigens. This flexibility should also prove helpful if future vaccines are modified to target specific variants, as our test can be modified to best assess their effectiveness in eliciting a NAb response.

These experiments also show that NAb measurement utilizing whole Spike protein led to consistently higher cross-neutralization to BA.1 and BA.2 than when the RBD antigen was used. This indicates that utilization of RBD renders the test more variant-specific. This may be due to a greater number of deleted epitopes from the omicron RBD protein when compared to the Spike protein such that the larger Spike protein has more binding epitopes available for polyclonal NAb to interfere with ACE-2 binding. The observed increase in ACE-2 binding to the BA.1 antigen in the 3 bead preparation (resulting in an apparent “negative inhibition”) was unexpected, and may be due to conformational changes of the antigen on the bead. Similar results were seen with a second preparation (data not shown). Given the large number of omicron breakthrough infections, even with relatively high levels of WT NAbs, our results indicate the RBD-multiplex platform is more clinically relevant than whole Spike protein-multiplex platform due to its increased variant-specificity. This is illustrated directly with the longitudinal case studies presented above in **Figure 4**. At the time of their breakthrough infections, all four subjects presented with robust NAbs to wildtype, moderate NAbs to spike BA.1 and BA.2, and virtually no NAb activity to RBD BA.1 and BA.2 (thus the RBD NAbs better predicted their vulnerability to omicron infection).

In this study, we observed steeper increases in NAb levels against BA.1 and BA.2 in vaccinated individuals who experienced a breakthrough infection than individuals who received a booster. This finding may not be too surprising since current boosting protocols utilize the original vaccine that was developed for protection against the WT virus. We also noted that vaccinated individuals showed maximal levels of NAb to WT and the BA.1 variant of SARS-CoV-2 following a breakthrough infection during the post-omicron period. In contrast, individuals who experienced a breakthrough infection prior to prevalence of the omicron variants only showed maximal levels of NAb to WT virus. Collectively, the increase in NAb activity against the WT and omicron variants may ease concerns about the antigenic imprinting phenomenon, which has been postulated as a potential hindrance for effectiveness of future vaccines in individuals with existing immunity.

Our multiplex platform allows for simultaneous assessment of multiple antibody isotypes (IgG, IgM, and IgA) to multiple viral antigens (in this instance, RBD and/or Spike from the different variants). This more comprehensive assessment of humoral immune status provides complementary information and a context for the NAb data. Across all cohorts and variants, neutralizing activity correlated strongly but not completely with IgG. Additionally, IgM antibodies have been shown to be neutralizing, though at the time points assessed in these experiments, IgM responses were seen almost exclusively in the convalesced (unvaccinated) cohort. Neither a vaccine booster nor a breakthrough infection increased IgM levels, consistent with its expected role in the initial primary but not secondary, longer-term humoral response. Interestingly, the increased IgA levels seen after a breakthrough infection (but not after a booster) may suggest another dimension in humoral immunity (e.g. mucosal immunity) that is elicited following natural infection. This IgA response may be part of the reason why the “hybrid immunity” in individuals after a breakthrough infection tends to be more robust than either natural or vaccine-induced immunity alone.

Since these experiments were performed, the omicron subvariant B.2.12.1 became predominant in the U.S., with BA.4 and BA.5 spreading rapidly. Early evidence suggests that these variants escape antibodies directed against the WT strain to an even greater degree than BA.1/BA.2; data showing whether they may evade antibodies elicited by a BA1/BA2 infection is still preliminary (20, 21). Continued surveillance of these and future variants of concern and incorporation of those that demonstrate significant lack of cross-neutralization into the test system are important priorities.

Limitations of this study include the limited clinical information associated with some of the samples and relatively small sample sizes. Also, the lack of sequencing data to objectively verify which subvariant were responsible for each infection limits interpretation of possible differences between BA.1 and BA.2.

Here we demonstrate the utility of a new platform for assessment of NAb responses to SARS-CoV-2 variants of interest and variants of concern to provide clinically meaningful data pertaining to immune status *vis a vis* the variant. Rapid, easily accessible tests that allow individuals to monitor their immune status against relevant variants should aid decisions about the appropriateness and cadence of vaccine boosters and other exposure mitigation strategies.

## Data availability statement

The raw data supporting the conclusions of this article will be made available by the authors, without undue reservation.

## Ethics statement

The studies involving human participants were reviewed and approved by WCGIRB. The patients/participants provided their written informed consent to participate in this study.

## Author contributions

HL, GC, and SS contributed to the conception and design of the study, HL performed the experiments and some statistical analysis. SV performed statistical analysis and wrote the first draft of the manuscript. All authors contributed to the article and approved the submitted version.

## Funding

All work described in this manuscript was funded by Aditxt, Inc.

## Conflict of interest

All authors are employees or stockholders of Aditxt, Inc., which performs the tests described.

## Publisher’s note

All claims expressed in this article are solely those of the authors and do not necessarily represent those of their affiliated organizations, or those of the publisher, the editors and the reviewers. Any product that may be evaluated in this article, or claim that may be made by its manufacturer, is not guaranteed or endorsed by the publisher.

## Supplementary material

Supplementary Figure 1 Variant-specific RBD IgM **(A)** and RBD IgA **(B)** antibodies were determined in 6 cohorts: 1) COVID-naïve non-vaccinated negative controls collected before the beginning of the pandemic [Negative (Group 1)], 2) negative controls collected during 2021 [Negative (Group 2)], 3) a group of convalesced subjects with predominantly mild symptoms [Convalesced (Group 1)], 4) a second group of convalesced subjects from Stanford Blood Center [Convalesced (Group 2)], as well as groups of vaccinated (Vaccinated), and vaccine boosted (Boosted) individuals. Only the convalesced (Group 2) displayed robust increase in IgM and IgA, which were almost undetectable to the beta, BA.1, and BA.2 RBD antigen.

Supplementary Figure 2 Comparison of antibody isotype levels to WT, BA.1, BA.2 RBD after breakthrough infection. A subset of vaccinated individuals experienced breakthrough infections during the course of the study. Breakthrough infections were separated into those likely to be pre-omicron (symptom onset before Dec 5, 2021, N=6) and those likely to be omicron (symptom onset after Dec 30, 2021, N=11). Breakthrough infections significantly increased IgG and IgA **(A–C, G–I)** (p<0.0001), but not IgM **(D–F)** levels. There were no differences between pre-omicron and omicron-era breakthroughs.

Supplementary Table 1 Cohort Demographics (see Methods for details)

Supplementary Table 2 F statistics from 2 way ANOVAs showing significant Cohort x Variant interactions. The “bead set” refers to either the preparation of beads with 8 different variant-specific RBD antigens (WT, alpha, beta, delta, epsilon, gamma, and omicron BA.1 and BA.2) or with 3 - either RBD or Spike antigen from WT, BA.1, and BA.2.
