# Supplementary figures and images for: Simultaneous measurement of multiple variant-specific SARS-CoV-2 neutralizing antibodies with a multiplexed flow cytometric assay
Source: Front Immunol. 2022 Nov 25;13:1039163. doi: 10.3389/fimmu.2022.1039163 (PMC9732243; doi:10.3389/fimmu.2022.1039163)

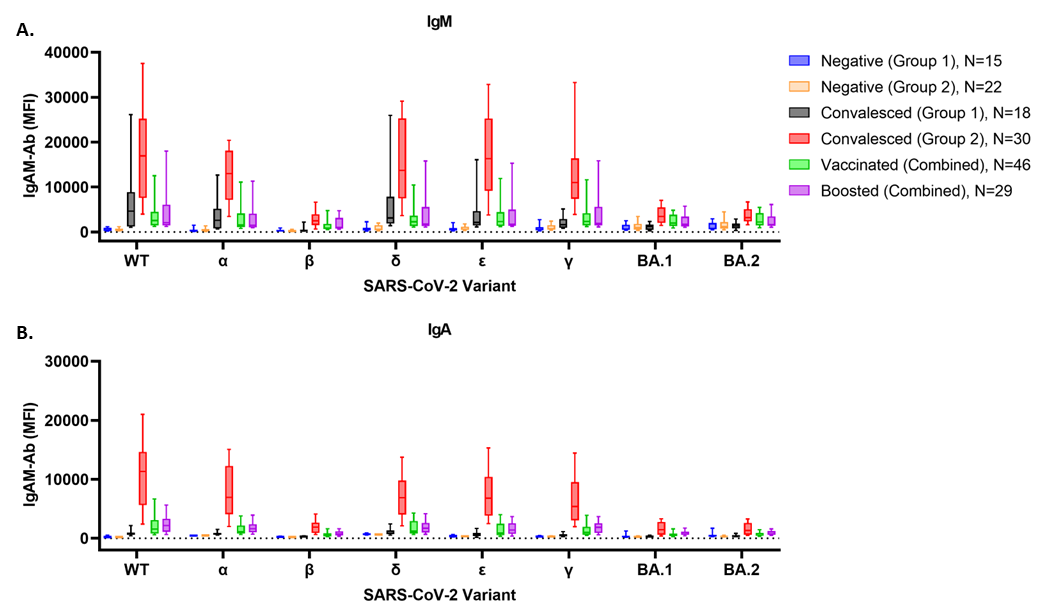

Supplement: Supplementary Figure 1 — Variant-specific RBD IgM (A) and RBD IgA (B) antibodies were determined in 6 cohorts: 1) COVID-naïve non-vaccinated negative controls collected before the beginning of the pandemic [Negative (Group 1)], 2) negative controls collected during 2021 [Negative (Group 2)], 3) a group of convalesced subjects with predominantly mild symptoms [Convalesced (Group 1)], 4) a second group of convalesced subjects from Stanford Blood Center [Convalesced (Group 2)], as well as groups of vaccinated (Vaccinated), and vaccine boosted (Boosted) individuals. Only the convalesced (Group 2) displayed robust increase in IgM and IgA, which were almost undetectable to the beta, BA.1, and BA.2 RBD antigen. [file Image_1.tif]

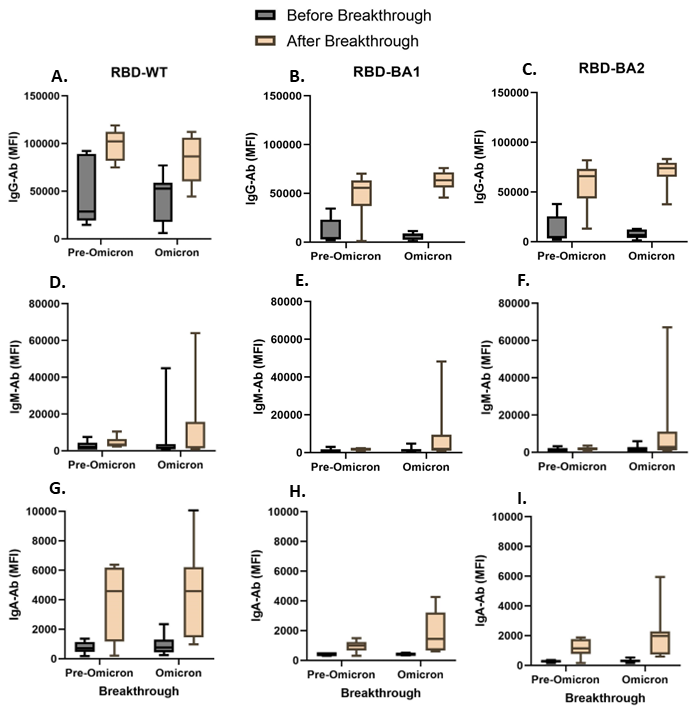

Supplement: Supplementary Figure 2 — Comparison of antibody isotype levels to WT, BA.1, BA.2 RBD after breakthrough infection. A subset of vaccinated individuals experienced breakthrough infections during the course of the study. Breakthrough infections were separated into those likely to be pre-omicron (symptom onset before Dec 5, 2021, N=6) and those likely to be omicron (symptom onset after Dec 30, 2021, N=11). Breakthrough infections significantly increased IgG and IgA (A–C, G–I) (p<0.0001), but not IgM (D–F) levels. There were no differences between pre-omicron and omicron-era breakthroughs. [file Image_2.tif]
