# Supplementary material for: Simultaneous measurement of multiple variant-specific SARS-CoV-2 neutralizing antibodies with a multiplexed flow cytometric assay
Source: Front Immunol. 2022 Nov 25;13:1039163. doi: 10.3389/fimmu.2022.1039163 (PMC9732243; doi:10.3389/fimmu.2022.1039163)
Supplement: Supplementary Table 1 — Cohort Demographics (see Methods for details) [file Table_1.docx]

| **Characteristic** | **Negative**  **(group 1)** | **Negative**  **(group 2)** | **Convalesced**  **(group 1)** | **Convalesced (group 2)** | **Vaccinated** | **Boosted** |
| --- | --- | --- | --- | --- | --- | --- |
| **Sample Size**  **(N)** | 15 | 22 | 18 | 30 | 46 | 29 |
| **Age**  **(Mean / STD)** | N/A | 45 (16) | 50 (12) | N/A | 56 (14) | 58 (15) |
| **Sex**  **(% Female)** | N/A | 50% | 50% | N/A | 54% | 47% |
| **BMI**  **(Mean / STD)** | N/A | 26 (4) | 29 (6) | N/A | 28 (11) | 26 (2) |
| **Days Since Last Vax Dose**  **(Mean / STD)** | N/A | N/A | N/A | N/A | 38 (28) | 49 (41) |
| **Vax Type**  **(% Moderna)**  **(% Pfizer)** | N/A | N/A | N/A | N/A | 37%  63% | 47%  53% |
| **Days Since Symptom Onset** | N/A | N/A | 63 (22) | N/A | N/A | N/A |

**Supplemental Table 1. Cohort Demographics** (see Methods for details)
