# Supplementary material for: Simultaneous measurement of multiple variant-specific SARS-CoV-2 neutralizing antibodies with a multiplexed flow cytometric assay
Source: Front Immunol. 2022 Nov 25;13:1039163. doi: 10.3389/fimmu.2022.1039163 (PMC9732243; doi:10.3389/fimmu.2022.1039163)
Supplement: Supplementary Table 2 — F statistics from 2 way ANOVAs showing significant Cohort x Variant interactions. The “bead set” refers to either the preparation of beads with 8 different variant-specific RBD antigens (WT, alpha, beta, delta, epsilon, gamma, and omicron BA.1 and BA.2) or with 3 - either RBD or Spike antigen from WT, BA.1, and BA.2. [file Table_2.docx]

| **Test** | **Bead Set** | **Antigen** | **F statistic** | **P value** | **Figure** |
| --- | --- | --- | --- | --- | --- |
| **Nabs** | 8 | RBD | F (35, 1078) = 31.69 | P<0.0001 | Figure 1 |
| **IgG** | 8 | RBD | F (35, 1064) = 43.60 | P<0.0001 | Figure 1 |
| **IgM** | 8 | RBD | F (35, 1071) = 7.09 | P<0.0001 | Sup Fig 1 |
| **IgA** | 8 | RBD | F (35, 1071) = 21.71 | P<0.0001 | Sup Fig 1 |
| **Nabs** | 3 | RBD | F (6, 170) = 31.12 | P<0.0001 | Figure 2 |
| **Nabs** | 3 | Spike | F (6, 178) = 51.00 | P<0.0001 | Figure 2 |
| **IgG** | 3 | RBD | F (6, 176) = 40.77 | P<0.0001 | Figure 2 |
| **IgG** | 3 | Spike | F (6, 178) = 16.12 | P<0.0001 | Figure 2 |
| **IgM** | 3 | RBD | F (6, 176) = 9.85 | P<0.0001 | Figure 2 |
| **IgM** | 3 | Spike | F (6, 178) = 13.50 | P<0.0001 | Figure 2 |
| **IgA** | 3 | RBD | F (6, 176) = 25.61 | P<0.0001 | Figure 2 |
| **IgA** | 3 | Spike | F (6, 178) = 6.22 | P<0.0001 | Figure 2 |

**Supplemental Table 2. F statistics from 2 way ANOVAs showing significant Cohort x Variant interactions.** The “bead set” refers to either the preparation of beads with 8 different variant-specific RBD antigens (WT, alpha, beta, delta, epsilon, gamma, and omicron BA.1 and BA.2) or with 3 - either RBD or Spike antigen from WT, BA.1, and BA.2.
